# The Effect of Different Doses of Intravenous Dexmedetomidine on the Properties of Subarachnoid Blockade: A Systematic Review and Meta-Analysis

**DOI:** 10.2147/LRA.S288726

**Published:** 2020-12-15

**Authors:** Mohammad K Al Nobani, Mohammed A Ayasa, Tarek A Tageldin, Abduljabbar Alhammoud, Marcus Daniel Lance

**Affiliations:** 1 Hamad Medical Corporation, Doha, Qatar

**Keywords:** dexmedetomidine, spinal anesthesia, adjuvant medication, subarachnoid block, prolongation of spinal anesthetic

## Abstract

**Background:**

Dexmedetomidine is a sedative and analgesic medication which has gained an increased usage as an adjuvant to both general and regional anaesthesia in recent years. In this systematic review and meta-analysis, we examined the changes to the characteristics of subarachnoid block when accompanied with intravenous dexmedetomidine. Our aim is to evaluate the effects of different doses of intravenous dexmedetomidine on the sensory and motor blockade duration of a single shot spinal anaesthetic and the incidence of any associated side effects.

**Methods:**

We searched published randomized clinical trials (RCTs) from January 1992 to April 2019 that investigated the use of IV dexmedetomidine with spinal anaesthesia. After considering our inclusion and exclusion criteria, we included 15 RCTs with 985 patients. We analyzed the duration of sensory and motor blockade and the related adverse effects in relation to different doses of IV dexmedetomidine.

**Results:**

Intravenous dexmedetomidine, with loading dose of 1 mcg/kg, prolonged the sensory blockade duration of spinal anaesthesia by a mean difference of 49.6 min, P<0.001, and motor blockade duration by a mean difference of 44.7 min, P<0.001, while a loading dose of 0.5 mcg/kg prolonged the sensory blockade by a mean difference of 43.06 min, P<0.001, and motor blockade duration by a mean difference of 29.09 min, P<0.001. Dexmedetomidine-related side effects were higher in patients receiving larger doses; the incidence of bradycardia was higher (OR=3.53, P<0.001) and incidence of hypotension showed a 1.29 fold increase when compared to the control group (P=0.065).

**Conclusion:**

The administration of intravenous dexmedetomidine in conjunction with spinal anaesthesia can significantly prolong the duration of both sensory and motor blockade. The use of larger loading doses of dexmedetomidine was associated with a larger side-effect profile with minimal beneficial changes when compared to lower loading doses.

## Background

Dexmedetomidine is a centrally acting selective α-2 receptor agonist that has hypnotic and analgesic properties.^1^ Since its introduction into anaesthesia practice, it has been widely used as an adjuvant to both general and regional anaesthesia, both as a sedative and as part of multimodal analgesia models. In recent years, multiple randomized controlled trials (RCTs) have emerged studying the effect of dexmedetomidine via multiple routes on the properties of subarachnoid block.^2^,^3^

In 2013, Abdullah^4^ and his colleagues published a meta-analysis of 7 RCTs studying the effects of intravenous dexmedetomidine combined with spinal anaesthesia, showing clinically significant prolongation of sensory and motor blockade duration. However, in the past 6 years, multiple new RCTs have also investigated the same question.

In our systematic review and meta-analysis, we have included 985 patients in 15 RCTs to study the effect of different doses of intravenous dexmedetomidine on spinal anaesthesia in terms of sensory blockade duration, motor blockade duration and the incidence of related side effects, such as hypotension, bradycardia, respiratory depression and postoperative sedation.

## Methods

We followed the Preferred Reporting Items for Systematic Reviews and Meta-Analysis (PRISMA-P) guidelines and checklist (Supplementary Materials) for the preparation of this article.^5^

### Study Selection

We searched all published RCTs from Medical Literature Analysis and Retrieval System Online database (MEDLINE via Pubmed), Cochrane Database of Systematic Reviews, Google Scholar database and the Excerpta Medica database (EMBASE) from the period January 1992 to April 2019.

We used the following search terms in combination with “Dexmedetomidine” or “Medetomidine”: Regional, Spinal, Subarachnoid, Intrathecal, Intravenous, IV, Systematic.

We also manually cross-referenced previous relevant reviews and identified RCTs that met our inclusion criteria.

### Inclusion and Exclusion Criteria

We included RCTs that investigated the characteristics of a single shot subarachnoid blockade with the use of intravenous dexmedetomidine as a bolus and/or infusion.

We excluded RCTs that: (1) were non-English language articles, (2) were animal studies, (3) used intrathecal injections of dexmedetomidine, or non-intravenous routes of dexmedetomidine, (4) used dexmedetomidine sedation for other type of procedures, (5) compared dexmedetomidine with other drugs that might affect the properties of subarachnoid block, (6) did not use a placebo arm, (7) were unpublished trials.

### Data Collection

The quality of the articles were assessed using JADAD^6^ scoring methodology, and the risk of bias was assessed by two independent authors (MNO and MAY). Articles with JADAD score < 3 were excluded from the analysis, and then the following data was extracted: type and dosage of the local anaesthetic used, dexmedetomidine dosage, sensory block duration and onset, motor block duration and onset, time to first analgesia use, and dexmedetomidine-related side effects (hypotension, bradycardia, respiratory depression and post-operative sedation).

One randomized controlled trial^7^ was excluded by consensus among the three authors, based on the risk of outcome bias (duration of both sensory and motor block). Moreover, we failed to contact the corresponding author.

With regard to outcome, we analyzed the following data: sensory and motor block duration in relation to different intravenous dexmedetomidine doses and related side effects.

The data were recorded and checked for any discrepancies by the three authors and entered into a preformed data spreadsheet. The discrepancies were resolved by re-examining the articles’ data.

### Data Analysis

Open meta-analysis software using a random-effect model was used for the data analysis, including subgroups analysis. The standardized Mean Difference (SDM) was used for the continuous variables, whereas the Relative Risk (RR) and 95% confidence intervals were used for the dichotomous variables. I^2^ was used to check the statistical heterogeneity across the studies.

### Articles Demographic Characteristics

Tables 1–3 summarize the demographic characteristics of the RCTs.

**Table 1:** Demographic Characteristics of the RCTs

Articles: Author/Year	Country	JADAD Score	Surgery	Number of Pts	Local Anesthetic
Agrawal 2016^32^	India	3	Lower extremity	80	12.5 mg bupivacaine + 10 mcg fentanyl
Park 2014^23^	South Korea	4	TURP + TURBT	42	6 mg bupivacaine
Al-Mustafa 2009^10^	Jordan	5	TURP + TURBT + TVT	48	12.5 mg bupivacaine
Reddy 2014^11^	India	5	Lower extremity	75	15 mg of bupivacaine
Kaya 2010^12^	Turkey	5	TURP	75	15 mg of bupivacaine
Lee 2014^21^	South Korea	5	Lower extremity	60	12 mg of bupivacaine
Elcicek 2010^13^	Turkey	5	Not mentioned	60	22.5 mg ropivacaine
Jung 2013^14^	South Korea	5	Lower extremity	60	12 mg of bupivacaine
Chan 2016^32^	Canada	5	Total knee arthroplasty	40	12.75 mg bupivacaine + 10 mcg fentanyl
Tekin 2007^15^	Turkey	4	Lower extremity + lower abdominal	60	80 mg prilocaine
Harsoor 2013^16^	India	5	Infraumbilical surgery	50	12.5 mg bupivacaine
Hong 2012^17^	South Korea	5	TURB	51	7.5 mg bupivacaine
Dinesh 2014^18^	India	5	Inguinal hernia, hysterectomy, arthroscopy ACL tear repair	100	15 mg of bupivacaine
Kumari 2017^19^	India	5	Lower abdominal surgery	60	15 mg of bupivacaine
Rekhi 2017^20^	India	3	Lower extremity	60	15 mg ropivacaine
Kavya 2018^22^	India	5	Infra-umbilical surgery	75	12.5 mg bupivacaine

**Table 2 t0002:** Group Characteristics of the RCTs

Articles: Author/Year	Groups	Dex Arms	Bolus	Timing	Maintenance
Agrawal 2016^31^	2	Dex 0.25	0.25 mcg/kg over 15 min	After spinal	0.3 mcg/kg/hr
Park 2014^23^	3	Dex 0.5	0.5 mcg/kg over 10 min	After spinal	N
		Dex 1	1 mcg/kg over 10 min	After spinal	N
Al-Mustafa 2009^10^	2	Dex 1	1 mcg/kg over 10 min	After spinal	0.5 mcg/kg/hr
Reddy 2014^11^	3	Dex 0.5	0.5 mcg/kg over 10 min	Before spinal	N
Kaya 2010^12^	3	Dex 0.5	0.5 mcg/kg over 10 min	Before spinal	N
Lee 2014^21^	3	Dex 0.5	0.5 mcg/kg over 10 min	Before spinal	N
		Dex 1	1 mcg/kg over 10 min	Before spinal	N
Elcicek 2010^13^	2	Dex 1	1 mcg/kg over 10 min	After spinal	0.4 mcg/kg/hr for 60 min
Jung 2013^14^	3	Dex 0.25	0.25 mcg/kg over 10 min	After spinal	N
		Dex 0.5	0.5 mcg/kg over 10 min	After spinal	N
Chan 2016^32^	2	Dex 0.5	0.5 mcg/kg over 10 min	Before spinal	0.5 mcg/kg/hr
Tekin 2007^15^	2	Dex 1	1 mcg/kg over 10 min	After spinal	0.4 mcg/kg/hr for 50 min
Harsoor 2013^16^	2	Dex 0.5	0.5 mcg/kg over 10 min	Before spinal	0.5 mcg/kg/hr
Hong 2012^17^	2	Dex 1	1 mcg/kg over 5 min	Before spinal	N
Dinesh 2014^18^	2	Dex 1	1 mcg/kg over 10 min	After spinal	0.5 mcg/kg/hr
Kumari 2017^19^	2	Dex 1	1 mcg/kg over 10 min	Before spinal	0.6 mcg/kg/hr
Rekhi 2017^20^	2	Dex 1	1 mcg/kg over 10 min	After spinal	0.5 mcg/kg/hr
Kavya 2018^22^	3	Dex 0.5	0.5 mcg/kg over 10 min	Before spinal	0.5 mcg/kg over 60 min
		Dex 1	1 mcg/kg over 10 min	Before spinal	N

**Abbreviations:** Dex 0.5, arm with IV dexmedetomidine loading dose of 0.5mcg/kg; Dex1, arm with IV dexmedetomidine loading dose of 1mcg/kg.

**Table 3:** Outcomes of the RCTs

Articles	Block Characteristics		Dexmedetomidine-Related Adverse Effects			
Author/Year	Sensory Block Duration	Motor Block Duration	Hypotension	Bradycardia	Respiratory Depression	Postop Sedation
Agrawal 2016^31^	Y	Y	Y	Y	Y	N
Park 2014^23^	Y	Y	Y	Y	Y	Y
Al-Mustafa 2009^10^	Y	Y	Y	Y	N	N
Reddy 2014^11^	Y	Y	Y	Y	Y	Y
Kaya 2010^12^	Y	Y	Y	Y	Y	Y
Lee 2014^21^	Y	Y	Y	Y	Y	Y
Elcicek 2010^13^	Y	Y	Y	Y	Y	Y
Jung 2013^14^	Y	Y	Y	Y	Y	Y
Chan 2016^32^	N	N	Y	Y	Y	Y
Tekin 2007^15^	Y	Y	Y	Y	Y	Y
Harsoor 2013^16^	Y	Y	Y	Y	Y	N
Hong 2012^17^	Y	Y	Y	Y	Y	Y
Dinesh 2014^18^	Y	Y	Y	Y	Y	Y
Kumari 2017^19^	Y	Y	Y	Y	N	Y
Rekhi 2017^20^	Y	Y	Y	Y	Y	Y
Kavya 2018^22^	Y	Y	Y	Y	Y	Y

## Results

15 intermediate to high quality trials met our inclusion criteria^8–23^ and investigated the effects of intravenous dexmedetomidine on the properties of a single shot subarachnoid block.

The analysis included 985 patients divided equally between the intervention group and the placebo control group.

**Figure 1:**
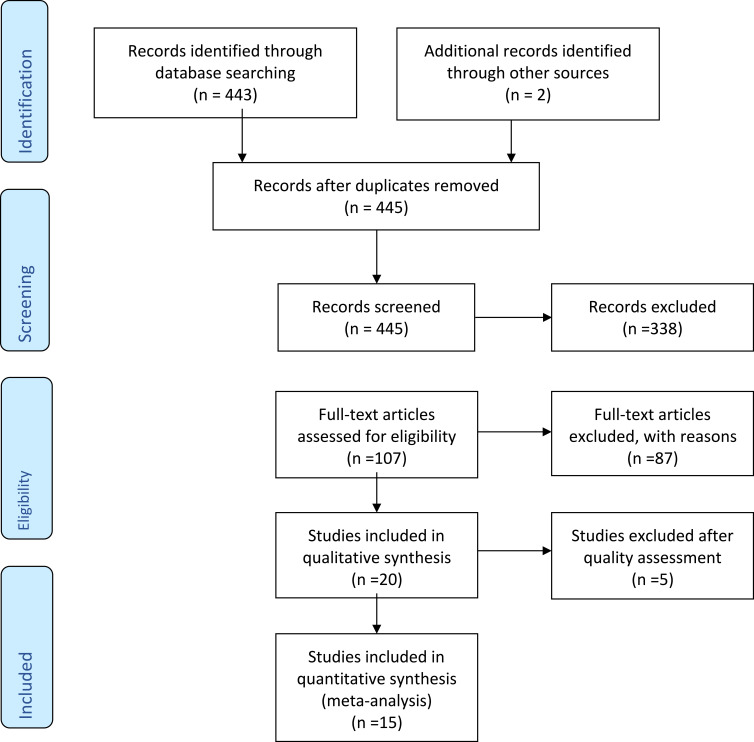
PRISMA flowchart.

**Note:** PRISMA figure adapted from Liberati A, Altman D, Tetzlaff J, et al. The PRISMA statement for reporting systematic reviews and meta-analyses of studies that evaluate health care interventions: explanation and elaboration. *Journal of clinical epidemiology*. 2009;62(10). Creative Commons.^24^

Figure 1, a PRISMA flow chart, summarizes the results of the screened, excluded and included RCTs in the final analysis.

Tables 1, [Table t0002]–3 show the demographic characteristics and outcomes retrieved from the included trials.

### Sensory Block Duration

Of the 15 RCTs we analyzed, four of the trials had 2 interventional arms of different doses of dexmedetomidine, making a total of 19 interventional arms.

Our analysis showed that administration of intravenous (IV) dexmedetomidine in conjunction with spinal anaesthesia significantly prolonged the duration of the sensory blockade regardless of the administered dose of dexmedetomidine or the type and dose of the used local anesthetic utilized, by a mean difference of 47.583 min, 95% CI (33.133–62.033), P<0.001, I^2^ = 95.7%.

Subgroup analysis of the duration of the blockade using IV dexmedetomidine 1 mcg/kg loading dose, showed prolongation of the duration of the block by a mean difference of 49.6 min, 95% CI (24.7–74.5), P<0.001, I^2^ = 97.3%. While using IV dexmedetomidine 0.5 mcg/kg loading dose, the duration was prolonged by a mean difference of 43.06 min, 95% CI (27.8–58.2), P<0.001, I^2^ = 83.9%.

**Figure 2:**
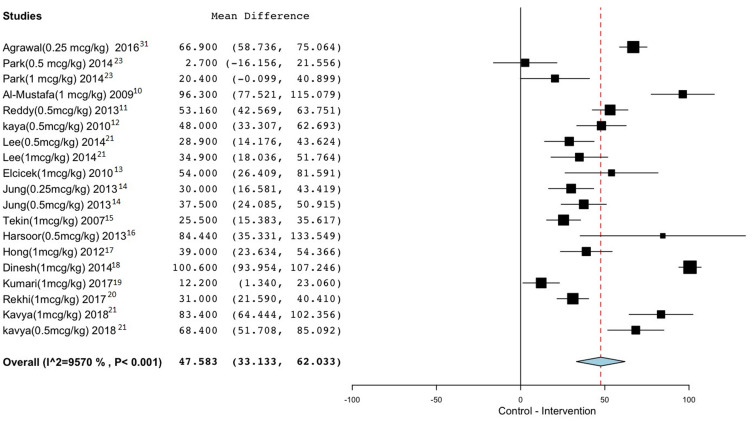
Sensory block duration.

Figure 2 and Table 4 summarize these findings.

**Table 4:** Results for Sensory and Motor Block Duration

Outcome	N of Dex Arms	N of Patients	Mean Difference (95% Confidence Interval)	P value for Statistical Significance	I^2^ Test for Heterogeneity
**Sensory Block Duration overall**	19	985	47.5 (33.13–62.03)	<0.001	95.7%
Dex 1mcg/kg	10	558	49.6 (24.7–74.5)	<0.001	97.3%
Dex 0.5mcg/kg	7	307	43.06 (27.8–58.2)	<0.001	83.9%
Dex 0.25mcg/kg	2	120	48.8 (12.6–85)	0.008	95.2%
**Motor Block Duration overall**	19	985	43.2 (23.6–62.7)	<0.001	97.5%
Dex 1mcg/kg	10	558	44.7 (23.2–66.3)	<0.001	97.03%
Dex 0.5mcg/kg	7	307	29.09 (9.3–48.7)	0.004	83.8%
Dex 0.25mcg/kg	2	120	83.6 (35.3–202.5)	0.168	99.03%

### Motor Block Duration

Of the 15 RCTs we analyzed, four of the trials had 2 interventional arms of different doses of dexmedetomidine, making a total of 19 interventional arms.

The IV administration of dexmedetomidine showed prolongation of motor blockade duration by a mean difference of 43.2 min, 95% CI (23.63–62.77), P<0.001, I^2^ = 97.5% regardless of the amount of administered loading dose of dexmedetomidine or type and dose of local anesthetic used.

Subgroup analysis using IV dexmedetomidine 1 mcg/kg loading dose showed prolongation of the duration of the blockade by a mean difference of 44.7 min, 95% CI (23.2–66.3), P<0.001, I^2^ = 97.03%. While using IV dexmedetomidine 0.5 mcg/kg loading dose, the duration was prolonged by a mean difference of 29.09 min, 95% CI (9.3–48.7), P=0.004, I^2^ = 83.8%.

**Figure 3:**
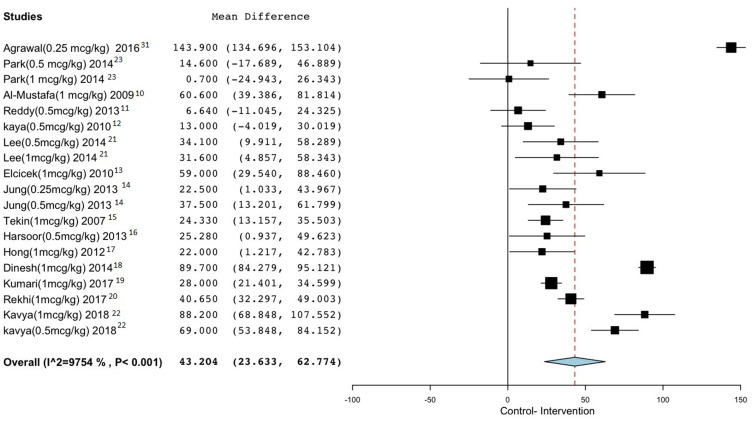
Motor block duration.

Figure 3 and Table 4 summarize these findings.

### Dexmedetomidine-Related Adverse Effects

Since there was an absence of any specific standard definition of dexmedetomidine-related adverse effects, we present the results as reported in the RCTs.

Table 5 summarizes the findings.

**Table 5:** Dexmedetomidine-Related Adverse Effects

Outcome	N of Dex Arms	N of Patients	Odds Ratio (95% Confidence Interval)	P value for Statistical Significance	I^2^ Test for Heterogeneity
**Bradycardia overall**	20	1025	3.53 (2.48–5.02)	<0.001	98.6%
Dex 1mcg/kg	10	558	4.72 (2.72–6/7)	<0.001	85.4%
Dex 0.5mcg/kg	8	347	2.81 (1.53–5.15)	<0.001	99.4%
Dex 0.25mcg/kg	2	120	1.74 (0.39–7.69)	0.462	70.5%
**Hypotension overall**	20	1025	1.29 (0.98–1.69)	0.065	12.03%
Dex 1mcg/kg	10	558	1.3 (0.78–2.15)	0.301	28.29%
Dex 0.5mcg/kg	8	347	1.12 (0.71–1.77)	0.616	9.5%
Dex 0.25mcg/kg	2	120	1.5 (0.52–4.5)	0.438	0%

#### Bradycardia

We analyzed 15 RCTs with a total of 19 interventional arms, having 985 patients in all arms.

The probability of bradycardia was higher in the patients receiving dexmedetomidine regardless of the dose administered (RR=3.57; 95% (2.48–5.12); P<0.001, I^2^ = 0%).

Subgroup analysis using IV dexmedetomidine 1 mcg/kg loading dose showed a higher probability of developing bradycardia (RR=4.27; 95% (2.72–6.7); P<0.001, I^2^ = 0%). While using IV dexmedetomidine 0.5 mcg/kg loading dose showed a higher probability of bradycardia compared to placebo (RR=2.77; 95% CI: (1.42–5.39), P<0.003, I^2^ = 0%)

There were no reported cases of prolonged or delayed bradycardia in the studied RCTs.

#### Hypotension

The relative risk of hypotension in the interventional group was 1.23-fold higher compared to the control group, regardless of the administered dose of dexmedetomidine (95% CI: 0.86–1.77; P=0.247, I^2^ =16.61%). Table 5 summarizes the subgroup analysis results.

#### Other Related Adverse Effects

Respiratory depression was reported in 15 RCTs. 13 cases were reported in the interventional group and 9 cases in the control group. There was no statistical significance noted between both arms.

Postoperative sedation was reported in 10 arms among 6 RCTs. The group that received dexmedetomidine was 5.86 times more likely to develop postoperative sedation (95% CI [3.35–10.25]; p value <0.001; I^2^ =0%). It should be noted that higher doses of dexmedetomidine were associated with a higher relative risk of postoperative sedation, loading dose of 1mcg/kg was 7 times more likely to be associated with postoperative sedation while loading dose of 0.5mcg/kg was 5.66 times higher compared to the control group (p value <0.001; I^2^ =0% for both).

## Discussion

The result of this review confirms the outcome of a smaller previously published systematic review,^4^ that the administration of IV dexmedetomidine in patients receiving subarachnoid blockade prolongs the duration of sensory and motor block. Higher doses (1mcg/kg loading dose) of dexmedetomidine were associated with a longer duration of both sensory and motor block when compared to lower doses of dexmedetomidine (0.5 mcg/kg loading dose or less), but also with a higher incidence of bradycardia and postoperative sedation. It should be noted that loading doses with 1mcg/kg dexmedetomidine when compared to 0.5mcg/kg or 0.25mcg/kg did not show a large difference in sensory block duration, while motor block duration was more significantly prolonged. There was no significant difference in the incidence of hypotension or respiratory depression between the IV dexmedetomidine group and the placebo-controlled group.

As an adjuvant medication, dexmedetomidine has been used by different routes to prolong the duration of local anaesthetics. It has been shown to prolong the duration of regional blocks when administered perineurally^25^,^26^ and shown to prolong the duration of subarachnoid block when administered via the intrathecal route,^2^,^27–29^ suggesting both peripheral and central mechanisms of action for dexmedetomidine. It has high selectivity towards α2-adrenergic receptors^30^ acting at the presynaptic C-fibers, postsynaptic dorsal horn neurons and locus ceruleus of the brain stem.^31^

There are several limitations in this review. We have included multiple RCTs looking into different outcomes as well as using different protocols of dexmedetomidine administration. Some studies used only a loading dose of dexmedetomidine while others followed it with maintenance infusions ranging from 0.2–0.6 mcg/kg/hr lasting for different durations. Some protocols continued the infusion until the end of surgery, while others infused the maintenance dose for a specific duration. Moreover, loading doses were administered over different durations, ranging from 5−15 minutes and were administered at different times in relation to the spinal anaesthetic injection. A small number of RCTs began the loading dose before the spinal injection, while others started it after establishment of the subarachnoid block.

In addition, different local anesthetic drugs with variable doses, were used for the spinal anaesthesia. One trial used prilocaine,^15^ two trials used ropivacaine^13^,^20^ while the remaining trials have used different doses of bupivacaine. Intrathecal fentanyl was used in two trials as an adjuvant to the local anesthetic.^32^,^33^

Finally, the end point for sensory block duration was defined differently in the trials, as some of them used time for two segment regression to cold or to pinprick sensation, while others did not specifically define how they assessed the sensory block duration. The absence of a standardized method of assessment was also observed when reporting the motor block duration and dexmedetomidine-related adverse effects were not clearly elucidated in all the clinical trials.

## Conclusion

We conclude that the administration of intravenous dexmedetomidine in conjunction with spinal anaesthesia can significantly prolong the duration of both sensory and motor blockade. Considering both advantages and disadvantages, the use of 1mcg/kg loading dose of dexmedetomidine was associated with a larger side-effect profile, while the beneficial changes to the characteristics of the subarachnoid blockade were minimal when compared to lower loading doses. In that sense, a lower loading dose should be preferred.

## Abbreviations

RCT, Randomized controlled trials; IV, intravenous; PRISMA-P, Preferred Reporting Items for Systematic Reviews and Meta-Analysis; SDM, Standardized mean difference; RR, Relative Risk; Mcg, micrograms; Kg, kilogram.

## Data Sharing Statement

Data material is available upon request.
